# Brush, byte, and bot: quality comparison of artificial intelligence-generated pediatric dental advice across ChatGPT, Gemini, and Copilot

**DOI:** 10.3389/froh.2025.1652422

**Published:** 2025-08-15

**Authors:** Deepika Kapoor, Deepanshu Garg, Santosh Kumar Tadakamadla

**Affiliations:** ^1^ Department of Pedodontics and Preventive Dentistry, Luxmi Bai Institute of Dental Sciences, Baba Farid University of Health Sciences, Punjab, India; ^2^ Department of Oral Medicine and Radiology, Luxmi Bai Institute of Dental Sciences, Baba Farid University of Health Sciences, Punjab, India; ^3^ Rural Dental and Oral Health Clinical Teaching School, La Trobe Rural Health School, Bendigo, Victoria, VIC, Australia

**Keywords:** artificial intelligence, pediatric dentistry, parental guidance, ChatGPT, Google Gemini, Microsoft Copilot, response quality

## Abstract

**Introduction:**

Artificial intelligence (AI) tools such as ChatGPT, Google Gemini, and Microsoft Copilot are increasingly relied upon by parents for immediate guidance on pediatric dental concerns. This study evaluated and compared the response quality of these AI platforms in addressing real-world parental queries related to pediatric dentistry, including early tooth extraction, space maintenance, and the decision to consult a pediatric or a general dentist.

**Methods:**

A structured 30-question survey was developed and submitted to each AI model, and their responses were anonymized and assessed by pediatric dental experts using a standardized rubric across five key domains: clinical accuracy, clarity, completeness, relevance, and absence of misleading information.

**Results:**

Statistically significant differences were found across all five domains (*p* < .001), with ChatGPT consistently achieving the highest scores. Multivariate analysis (MANOVA) confirmed a strong overall effect of the AI model on response quality (Pillai's Trace = 0.892, *p* < .001), supporting ChatGPT's superior performance in providing accurate, relevant, and comprehensive pediatric dental advice.

**Discussion:**

While AI technologies show potential as clinical decision support systems, their variable performance reinforces the need for expert oversight. Future AI development should focus on optimizing response quality and safety to ensure effective and trustworthy digital health communication for pediatric dental care.

## 1 Introduction

Artificial Intelligence (AI) is revolutionizing modern healthcare, reshaping and redefining how patients and professionals interact with clinical information, decision-making tools, and therapeutic guidance. Over the last decade, the implementation of AI in dentistry has attracted significant interest from dental practitioners, particularly in diagnostic imaging, treatment planning, and patient communication (1). With the rapid advancement of natural language processing (NLP) models, a new dimension of AI application has emerged: real-time conversational support for health-related queries. AI platforms such as ChatGPT, Microsoft Copilot, and Google Gemini are increasingly being used not only by clinicians but also by laypersons particularly caregivers seeking instant information regarding their children's health (2).

In pediatric dentistry, parental involvement and health literacy are essential to ensure timely, appropriate oral care. Queries such as whether a child's decayed tooth needs extraction, when to use a space maintainer, or whether to visit a general dentist or a specialist for a specific condition, are routinely raised by parents. Traditionally, parents used their routine consultations with health practitioners to address such queries. With the widespread availability of internet access, individuals initially turned to platforms like Google and YouTube for health-related information; some of which proved informative, while others were misleading (3, 4). The recent rise of AI-driven assistants has further shifted these interactions to intelligent digital platforms, prompting an urgent need to assess the safety, reliability, and clinical efficacy of AI-generated responses (5).

Moreover, underserved and rural populations often rely on online information due to geographic and resource constraints, increasing their vulnerability to AI-generated misinformation. Evaluating the accuracy, clarity, and safety of chatbot responses in pediatric dentistry is therefore not only academically relevant but also crucial for equitable and reliable public health communication. Despite the integration of AI in health information portals and chatbots, there remains limited empirical evidence on how these tools perform specifically in pediatric dental scenarios (6).

Previous studies have also explored the use of AI chatbots in dental education, particularly in enhancing student engagement, knowledge acquisition, and communication skills. For instance, Uribe et al. conducted a global survey among dental educators and found cautious optimism about integrating AI chatbots like ChatGPT into dental curricula, emphasizing the need for clear guidelines and training to maximize educational value while mitigating risks of misinformation and reduced human interaction (7). Similarly, Or et al. implemented a chatbot for improving patient history-taking skills in dental students and observed increased participation and perceived competence, although concerns about generative accuracy were noted (8). A recent educational intervention at the University of Illinois Chicago College of Dentistry (UIC-COD) compared a traditional learning management system (Blackboard) with a rule-based chatbot designed to guide predoctoral students through clinical implant protocols. The chatbot significantly improved students' engagement, convenience, and interaction with content while reducing faculty workload and student anxiety. Although no significant difference in perceived accuracy was observed between the two platforms, students preferred the chatbot for its real-time, accessible, and interactive support, highlighting the broader pedagogical value of AI tools in dental education (9).

A few recent studies have explored the ability of AI chatbots to answer dental board examination questions with varying degrees of accuracy. For instance, Chau et al. evaluated GPT-4.0, Claude-2, and Llama-2 on prosthodontic and restorative dentistry MCQs from INBDE and ORE exams, focusing on answer accuracy and rationale quality (10). Chao et al. evaluated ChatGPT-3.5 and 4.0 using 1461 MCQs from the US and UK dental licensing exams, finding that ChatGPT-4.0 exceeded the passing thresholds while 3.5 fell short, demonstrating improved capabilities in written examination settings (11). Jung et al. conducted a focused analysis on pediatric dentistry questions from the Korean National Dental Board Exam and reported that the AI models underperformed compared to acceptable competency levels (12).

While these investigations highlight AI's emerging role in academic assessment environments, our study diverges by testing chatbot-generated responses to real-world caregiver questions in pediatric dentistry. This approach allows us to evaluate AI output from a patient-facing communication and safety perspective, moving beyond academic correctness to practical relevance, completeness, and clinical clarity which is very crucial for the applicability and potential benefit to rural and underserved communities. This specialty demands not only clinical accuracy but also developmentally appropriate, parent-friendly communication especially when guiding caregivers who may have limited access to pediatric dental specialists.

Some of the recent studies have begun to explore the application of AI in pediatric dentistry, particularly in areas such as caries detection, image segmentation, and diagnostic planning. For instance, a scoping review by Schwendicke et al. (2020) identified promising AI-based approaches in early diagnosis and treatment planning, though it emphasized the need for further validation through robust clinical studies (13). Similarly, Rokhshad et al. (2023) demonstrated that AI tools like ChatGPT and Gemini can support literature screening tasks in pediatric dentistry with high sensitivity, yet human oversight remains essential to ensure accuracy (14). In another study, convolutional neural networks achieved over 97% accuracy in detecting anatomical landmarks on pediatric panoramic radiographs, underscoring the potential of AI in enhancing clinical decision-making (15).

The complexity of pediatric dentistry, including behavior management, growth-related decision-making, and psychological sensitivity, makes it critical that any digital aid including AI communicates not just information, but also clarity, empathy, and tailored guidance. A misinformed or unclear AI response could lead to delayed care, inappropriate decisions, or undue anxiety.

This study is novel in its comparative evaluation of the responses to parents' real-life questions obtained from three leading AI models using a structured questionnaire. Therefore, this study aims to critically evaluate the clinical accuracy, relevance, clarity, completeness, and potential for misinformation in AI-generated responses from ChatGPT (4.0), Microsoft Copilot, and Google Gemini to common parental queries in pediatric dentistry. By assessing the safety, reliability, and efficacy of these platforms, the study seeks to inform both healthcare professionals and AI developers about their current utility and limitations in real-world pediatric dental communication (16).

## 2 Materials and methods

### 2.1 Study design

This study employed a cross-sectional design aimed at evaluating the clinical accuracy, relevance, clarity of language, completeness of information, and presence or absence of misleading content in AI-generated responses to pediatric dental queries.

### 2.2 Questionnaire development

The 30-item questionnaire was developed by a panel of senior pediatric dental faculty members based on real-life parental concerns frequently encountered during clinical consultations over the past year. The questionnaire was also supplemented with questions frequently encountered on publicly accessible platforms such as the American Academy of Pediatric Dentistry (AAPD) FAQ section and the WebMD Dental Health section. This ensured the inclusion of frequently queried, real-world parental concerns and enhanced the relevance and generalizability of the survey content (17, 18).

A preliminary pool of questions was compiled prior to applying the inclusion and exclusion criteria to derive the final 30-item questionnaire. Inclusion criteria for selecting questions included frequency of occurrence, clinical relevance to pediatric oral health decision-making, and suitability for evaluation by AI models.

Questions that were vague, redundant, or too case-specific were excluded following expert consensus. To ensure content validity, the questionnaire was independently reviewed by three subject matter experts in pediatric dentistry. Their feedback guided revisions to improve clarity, remove ambiguity, and ensure broad applicability. While a formal psychometric pilot study was not conducted, this expert-driven review process served as a practical validation approach grounded in clinical experience. The finalized questionnaire and the expert evaluation rubric have been provided as Supplementary Material.

The questions encompassed key areas of parental concern in pediatric dentistry, including the appropriate timing and necessity of extracting primary teeth, the use and implications of space maintainers, decision-making around whether to consult a pediatric dentist or a general practitioner, recommended preventive dental care and home hygiene practices, and commonly held myths and sociocultural beliefs related to children's oral health. The finalized questionnaire was administered identically to each of the three AI models (19):

ChatGPT (OpenAI) which was accessed via the GPT-4 interface using a subscribed version, Microsoft Copilot (free version) accessed through its integration with Microsoft Office applications, and Google Gemini (free version) accessed via the Gemini web-based platform.

All responses were generated between 15.04.2025 and 17.04.2025 using the same prompts under neutral, non-personalized settings to ensure consistency (20).

### 2.3 Evaluation rubric

An expert evaluation rubric was developed by the research team specifically for this study to assess AI-generated responses. The rubric was designed to capture key quality indicators including clinical accuracy, relevance to the question, clarity of language, completeness of information, and the absence of misleading information, each rated on a 5-point Likert scale where a score of 1 indicated the lowest performance (e.g., inaccurate, irrelevant, unclear, incomplete, or misleading), and a score of 5 indicated the highest performance in that criterion.

A panel of three pediatric dentists independently evaluated the AI-generated responses, with each response anonymized to prevent evaluator bias (21).

### 2.4 Inter-rater reliability assessment

To assess consistency among the three expert raters, inter-rater reliability was calculated using the Intraclass Correlation Coefficient (ICC). A two-way random-effects model (ICC2,1) with absolute agreement was used, based on 90 responses rated independently by all three pediatric dentistry experts. ICCs were computed separately for each evaluation domain—Accuracy, Relevance, Clarity, Completeness, and No Misleading Information. The analysis was conducted using JASP software (version 0.19.3.0) (22).

### 2.5 Statistical analysis

The individual scores from all three evaluators for each question were first summed and then averaged to generate a single composite score per domain for each AI response. This process ensured a unified score per AI model per question, incorporating input from all three evaluators while minimizing individual rater bias.

To compare performance across AI platforms, five separate one-way analyses of variance (ANOVAs) were conducted—one for each evaluation domain. These ANOVAs compared the mean domain scores (across all 30 questions) between the three AI models. When statistically significant differences were found (*p* < 0.05), Tukey's Honest Significant Difference (HSD) *post hoc* tests were performed to identify pairwise differences between AI platforms.

In addition, a multivariate analysis of variance (MANOVA) was conducted to assess the overall effect of AI model on the combined set of response quality measures (i.e., all five domains simultaneously). This multivariate approach was employed to detect any overarching patterns or interactions that might not be apparent in individual domain analyses. The assumption of homogeneity of covariance matrices was evaluated using Box's M test. As this test was significant, indicating violation of the assumption, Pillai's Trace was reported due to its robustness under such conditions. Effect sizes were calculated using omega squared (*ω*^2^), as it provides a less biased estimate of population-level variance explained in ANOVA designs compared to eta squared, particularly when sample sizes are equal or moderately sized.

All statistical analyses were performed using JASP statistical software (version 0.19.3.0) (22).

## 3 Results

This study evaluated and compared the responses of three AI models—ChatGPT, Google Gemini, and Microsoft Copilot—across five key dimensions of response quality: accuracy, relevance, clarity, completeness, and absence of misleading information. Both statistical analysis (ANOVA and MANOVA) of AI-generated answers were conducted to assess performance. Sample responses to representative pediatric dental queries are shown in Table 1, and they serve to illustrate the statistical trends described below.

**Table 1:** Sample AI responses to generic pediatric dental questions.

Questions	Chat Gpt 4.0	Google Gemini	Microsoft Pilot
1. Should I save my child's tooth or extract it if it's painful?	If possible, it's better to save the tooth. Pain can often be managed with treatment such as pulp therapy or a filling. Early extraction should only be considered if the tooth is non-restorable or poses a risk of infection.	It's generally recommended to save a child's tooth, if possible, even if it's painful, especially if it's a milk tooth that's not due to fall out soon.	Saving the tooth is generally preferred, if possible, as it helps maintain normal oral function and guides permanent teeth into place.
2. What is a space maintainer?	A space maintainer is a dental appliance used to preserve space for a permanent tooth after a premature loss of a baby tooth.	A space maintainer is a custom-made dental appliance used to hold open the space left by a prematurely lost milk tooth.	It is a dental appliance that holds space for a permanent tooth when a primary tooth is lost prematurely.
3. Should I visit a pediatric dentist or a general dentist for my child?	A pediatric dentist is specially trained for children's dental needs and behavior management. For complex or age-specific care, they are recommended.	For children, it is generally recommended to visit a pediatric dentist.	A pediatric dentist is specialized in children's dental care and behavioral management, making them a better choice.
4. How often should a child visit a dentist?	Every 6 months, or as advised by the dentist based on individual risk and needs.	A child should visit the dentist for a check-up every six months, starting from the eruption of their first tooth or by their first birthday, whichever comes first.	Every six months for routine check-ups and preventive care.
5. Will my child's permanent teeth come early if milk teeth are extracted?	No. The timing of permanent teeth eruption is determined by developmental biology, not by early extraction.	No, generally the opposite can happen. If milk teeth are extracted too early, the permanent teeth may actually be delayed or erupt in a misaligned position because the natural space maintainer is lost, and adjacent teeth can drift into the vacant spot.	Not necessarily. Eruption timing depends on developmental patterns.

### 3.1 Accuracy

**Figure 1:**
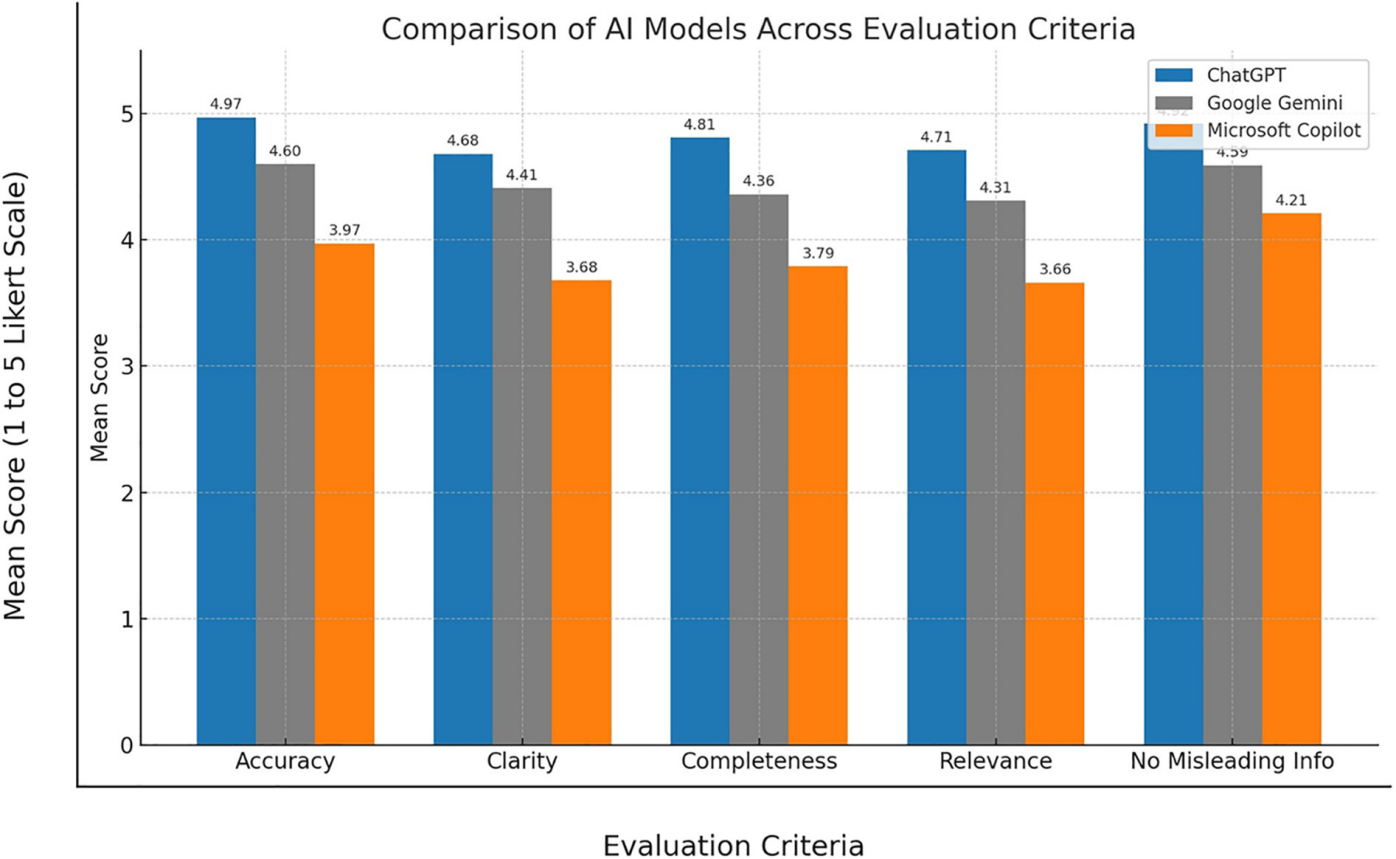
Combined bar graph of all variables across AI models.

A one-way ANOVA showed a significant difference in accuracy scores across the three AI models (*p* < .001). ChatGPT achieved the highest rating (*M* = 4.97, SD = 0.18), followed by Gemini (*M* = 4.60, SD = 0.49), and Copilot (*M* = 3.97, SD = 0.24) (Table 2) (Figure 1). For instance, in Table 1 (Q5), ChatGPT and Gemini accurately explained the biological nature of tooth eruption, while Copilot's response lacked clarity on the underlying mechanism.

**Table 2:** Summary of ANOVA results and mean scores.

Variable	*F*	*p*	*ω* ^2^	ChatGPT (Mean ± SD)	Gemini (Mean ± SD)	Copilot (Mean ± SD)
Accuracy	209.180	<.001	0.607	4.97 ± 0.18	4.60 ± 0.49	3.97 ± 0.24
Clarity	102.117	<.001	0.428	4.68 ± 0.47	4.41 ± 0.52	3.68 ± 0.47
Completeness	127.575	<.001	0.484	4.81 ± 0.39	4.36 ± 0.48	3.79 ± 0.41
No misleading info	57.141	<.001	0.294	4.92 ± 0.27	4.59 ± 0.49	4.21 ± 0.53
Relevance	113.549	<.001	0.455	4.71 ± 0.46	4.31 ± 0.49	3.66 ± 0.48

### 3.2 Relevance

Relevance scores also differed significantly (*p* < .001), with ChatGPT scoring highest (*M* = 4.71, SD = 0.46), followed by Gemini (*M* = 4.31, SD = 0.49) and Copilot (*M* = 3.66, SD = 0.48) (Table 2) (Figure 1). As shown in Table 1 (Q1), ChatGPT addressed treatment decision-making in detail, while Gemini provided a simpler yet appropriate response. Copilot's limited explanation reduced its relevance.

### 3.3 Clarity

There was a significant variation in clarity across models (*p* < .001). ChatGPT (*M* = 4.68, SD = 0.47) was rated clearest, followed by Gemini (*M* = 4.41, SD = 0.52), and Copilot (*M* = 3.68, SD = 0.47) (Table 2) (Figure 1). In Table 1 (Q2), while all three models defined the term “space maintainer,” ChatGPT used simpler phrasing and logical flow. Copilot's technical language made interpretation more difficult for lay readers.

### 3.4 Completeness

Completeness also varied significantly (*p* < .001). ChatGPT provided the most comprehensive answers (*M* = 4.81, SD = 0.39), with Gemini moderately complete (*M* = 4.36, SD = 0.48), and Copilot less so (*M* = 3.79, SD = 0.41) (Table 2) (Figure 1). For example, in Table 1 (Q3), ChatGPT included clinical reasoning, whereas Copilot's brief response lacked supporting detail.

### 3.5 No misleading information

The models differed significantly in avoiding misinformation (*p* < .001). ChatGPT achieved the highest safety rating (*M* = 4.92, SD = 0.27), followed by Gemini (*M* = 4.59, SD = 0.49) and Copilot (*M* = 4.21, SD = 0.53) (Table 2) (Figure 1). In Table 1 (Q5), Copilot's vague wording could imply incorrect timing of eruption post-extraction, while ChatGPT and Gemini more accurately clarified the biological timeline.

### 3.6 ANOVA and *post hoc* analysis

As presented in Table 2, one-way ANOVA revealed statistically significant differences across all five evaluative domains-Accuracy, Clarity, Completeness, No Misleading Information, and Relevance—among the three AI platforms (ChatGPT, Gemini, and Copilot), with *p*-values < .001 for all comparisons. The F-statistics ranged from 57.141 (No Misleading Information) to 209.180 (Accuracy), indicating robust between-group variance.

The associated omega squared (*ω*^2^) values, which reflect the proportion of variance explained by group differences, were substantial in all domains. Specifically, *ω*^2^ values ranged from 0.294 (No Misleading Information) to 0.607 (Accuracy), indicating moderate to large effect sizes suggesting that the differences in AI-generated responses were not only statistically significant but also practically meaningful in terms of their magnitude.

### 3.7 Inter-rater reliability results

The inter-rater reliability was assessed using Intraclass Correlation Coefficients (ICC2,1), assuming a two-way random effects model with absolute agreement. The ICC values for Accuracy (0.737), Clarity (0.689), Relevance (0.697), Completeness (0.857), and No Misleading Information (0.909) indicate moderate to excellent agreement among the three expert raters. These values support the consistency and objectivity of expert evaluations across all response components (Table 3).

**Table 3:** Inter-rater reliability among expert raters across evaluation components.

Component	ICC (2,1)	95% CI lower	95% CI upper	Interpretation
Accuracy	0.737	0.653	0.809	Good
Clarity	0.689	0.593	0.771	Moderate to good
Completeness	0.857	0.804	0.898	Excellent
No misleading info	0.909	0.874	0.936	Excellent
Relevance	0.697	0.604	0.777	Moderate to good

### 3.8 Multivariate analysis (MANOVA)

A MANOVA incorporating all five dimensions confirmed significant overall differences among the AI models: Pillai's Trace = 0.892, *p* < .001. Despite a significant Box's *M* test (*p* < .001), the use of Pillai's Trace—robust to such violations—supports the reliability of the results.

Post-hoc comparisons reaffirmed that ChatGPT consistently outperformed both Gemini and Copilot across all dimensions, especially in accuracy and clarity. Microsoft Copilot lagged behind significantly in completeness and relevance, limiting its effectiveness in pediatric dental guidance.

## 4 Discussion

This study offers a novel comparison of AI-generated responses to pediatric dental queries posed by parents and caregivers, highlighting the emerging role of conversational AI in facilitating patient communication. The observed performance variations among ChatGPT, Google Gemini, and Microsoft Copilot emphasize the importance of establishing evidence-based benchmarks for the use of AI tools in dentistry. Multivariate analysis revealed statistically significant distinctions across the five evaluation domains, with ChatGPT consistently outperforming the others particularly in clinical accuracy and clarity, two domains that are pivotal in guiding parental decision-making in pediatric dentistry.

Microsoft Copilot, in contrast, scored lower across all domains. This underperformance may be attributed to limitations such as insufficient domain-specific training, smaller or less diverse datasets, and increased sensitivity to prompt structure. These factors likely hindered the model's ability to generate coherent, context-appropriate, and clinically sound information. Google Gemini followed closely but often displayed generalized language and less nuanced clinical guidance. ChatGPT, by comparison, demonstrated strength in generating tailored and clinically relevant responses—likely due to its extensive training on large, diverse datasets and the incorporation of reinforcement learning with human feedback (RLHF) (23).

The MANOVA analysis further strengthened these findings by revealing significant multivariate differences among the models. Despite a violation of the homogeneity assumption (Box's *M* test), the robustness of Pillai's Trace confirmed the reliability of our statistical results. Importantly, our analysis incorporated both univariate (ANOVA) and multivariate (MANOVA) statistical approaches to provide a robust comparison across the five evaluation domains. This methodological rigor reduces bias and strengthens the credibility of our conclusions (21–23).

Our rubric-based evaluation, conducted by experienced pediatric dentists, ensured systematic scoring and minimized subjectivity. However, we acknowledge that our study assessed only one-time responses to standardized prompts, which does not capture the dynamic and iterative nature of real-world interactions with AI systems. As such, our findings may not fully reflect how these tools perform in longitudinal or interactive scenarios.

From a practical standpoint, AI tools like ChatGPT and Gemini demonstrate potential for meaningful integration into pediatric dental workflows (12, 15). These tools could support clinicians by generating parent-focused educational content, assisting in preliminary triage for remote consultations, and enhancing the communication of routine dental procedures (6, 8, 9, 24). Such integration may be particularly valuable in busy or underserved clinical settings, helping to reduce workload while improving caregiver understanding and compliance (25).

While AI holds great promise as a supportive tool in pediatric oral health, its current application should remain adjunctive rather than substitutive. Professional dental consultation must continue to serve as the gold standard for diagnosis and treatment (26). However, AI models can help bridge communication gaps in rural or underserved regions, function as educational resources, and provide immediate support provided their outputs are clinically reliable and validated through research such as this.

For instance, a recent systematic review demonstrated that AI-driven Clinical Decision Support Systems (CDSSs) enhance diagnostic accuracy and enable timely, patient-specific decision-making across healthcare environments. Moreover, the integration of AI-CDSSs has shown promise in reducing medication errors and optimizing treatment choices, ultimately contributing to improved patient outcomes (27). These findings reinforce the value of carefully vetted AI interventions in enhancing clinical decision-making paralleling the results observed in our pediatric dental study.

Additionally, evidence from recent reviews highlight that most conversational agents with unconstrained natural language input are still in early developmental stages, with limited evaluation of their efficacy or patient safety (27). The lack of robust experimental designs in many studies suggests a need for more rigorous trials to establish the clinical utility and safety profile of AI-based conversational tools in healthcare (27).

These outcomes are consistent with existing literature, which suggests that transformer-based models like GPT-4 offer superior contextual reasoning and empathy simulation compared to other AI formats (24). Emerging evidence also suggests broader applications of AI language models like ChatGPT across dental education, clinical management, and research. As noted by Puleio et al. (2024), ChatGPT has demonstrated utility across educational, administrative, and diagnostic domains within dentistry (28). Our findings contribute to this expanding literature, affirming ChatGPT's capabilities in patient-facing scenarios and underscoring the need for domain-specific evaluation protocols.

Nonetheless, the study did not explicitly examine ethical considerations such as the potential for AI-generated misinformation, data privacy risks, and the challenge of accountability. Responsibility for managing misinformation must be shared: developers and platforms should ensure ongoing model improvement and transparency regarding known limitations, while healthcare professionals and end-users must remain vigilant and avoid over-reliance on AI tools. Safeguards such as disclaimers, human oversight, and regulatory frameworks are essential to mitigate unintended harms in pediatric communication contexts. Future work should also assess how caregivers interpret and act upon AI-generated dental information. Incorporating outcomes such as parental satisfaction, confidence in decision-making, and actual behavior change will be critical in understanding the true impact of these tools (29).

This study has limitations. Notably, our 30-question instrument was not subjected to formal psychometric validation. Although questions were drawn from actual clinical encounters and reviewed by experts, future work should include validation steps such as reliability testing and factor analysis to enhance standardization and applicability. Another limitation is the inherent variability in large language model outputs. Despite using standardized, neutral prompts within a controlled timeframe, AI-generated responses may still vary due to internal model updates or prompt sensitivity. While we minimized such inconsistencies in our methodology, they remain an important consideration for reproducibility.

Future research should prioritize interactive, real-time assessments of AI tools in longitudinal caregiver contexts. Another valuable direction is the development of culturally and linguistically tailored AI models for pediatric oral health communication. Studies should also evaluate behavioral and comprehension outcomes among parents and caregivers exposed to AI-assisted dental guidance. Furthermore, exploring the role of AI in addressing gaps in access especially in rural or underserved populations can offer critical insights for scaling up digital health tools safely and effectively.

To conclude, the integration of AI into pediatric dental communication is a promising and rapidly evolving area. Among the tools evaluated, ChatGPT provided the most accurate, relevant, and comprehensible responses to caregiver queries, followed by Google Gemini and then Microsoft Copilot. While each model shows potential as an informational aid, their variability underscores the need for expert oversight and systematic validation. As AI technology continues to mature, its optimal use lies in complementing professional expertise particularly in sensitive fields like pediatric dentistry. Future efforts should focus on developing and implementing evidence-backed, context-specific, and ethically guided AI tools to ensure safe and effective support for caregivers and healthcare providers alike.

## Data Availability

The raw data supporting the conclusions of this article will be made available by the authors, without undue reservation.
