# Supplementary material for: Brush, byte, and bot: quality comparison of artificial intelligence-generated pediatric dental advice across ChatGPT, Gemini, and Copilot
Source: Front Oral Health. 2025 Aug 15;6:1652422. doi: 10.3389/froh.2025.1652422 (PMC12394529; doi:10.3389/froh.2025.1652422)
Supplement: Supplementary file 1 [file Table1.docx]

S1. AI Pediatric Dentistry Questionnaire

# Early Tooth Extraction & Management

1. Should I save my child’s tooth or extract it if it’s painful?
2. Is early extraction of a milk tooth harmful?
3. What happens if I delay visiting the dentist for a broken milk tooth?
4. Can an early extraction affect the permanent teeth?
5. How soon should I take my child to a dentist after trauma?

# Space Maintainers

1. What is a space maintainer?
2. Does every extracted tooth need a space maintainer?
3. What happens if I don’t put a space maintainer?
4. How long does a space maintainer need to stay?
5. Can my child eat normally with a space maintainer?
6. Are there different types of space maintainers?
7. How much does a space maintainer cost on average?
8. Is it painful to get a space maintainer?
9. Can space maintainers be avoided with early care?

# Treatment Decisions

1. Should I visit a pediatric dentist or a general dentist for my child?
2. What’s the difference between a general dentist and a pediatric dentist?
3. What treatments are exclusive to pediatric dentists?
4. Can milk teeth get root canals?
5. Are milk teeth even worth treating?
6. Is general anesthesia safe for dental procedures in kids?

# Preventive Care & Beliefs

1. How often should a child visit a dentist?
2. What’s the role of fluoride in milk teeth?
3. Are dental X-rays safe for children?
4. Is thumb sucking harmful for teeth?
5. Are cavities in milk teeth as serious as in permanent teeth?

# Myths & Social Attitudes

1. Will my child’s permanent teeth come early if milk teeth are extracted?
2. My child’s teeth are crooked—can early extraction help?
3. Should decayed teeth be removed early to avoid spreading infection?
4. Do milk teeth need cleaning and flossing?
5. Can diet alone keep milk teeth healthy?

**S 2. Expert Evaluation Rubric for AI Responses**

| Criterion | Definition | Scoring (1–5) |
| --- | --- | --- |
| Accuracy | Does the response contain factually correct dental information? | 1 = Completely inaccurate; 5 = Completely accurate |
| Relevance to the Question | Is the response directly related to the question asked? | 1 = Irrelevant; 5 = Directly relevant |
| Clarity of Language | Is the response written in an easily understandable and parent-friendly language? | 1 = Very unclear/confusing; 5 = Very clear |
| Completeness of Information | Does the response cover all important aspects of the question? | 1 = Incomplete; 5 = Thorough and comprehensive |
| Potential for Misleading Information | Does the response avoid misinformation or potentially harmful advice? | 1 = Highly misleading; 5 = No misleading information |
